# Comprehensive Analysis of Temperature-Dependent Photoluminescence in Silica-Encapsulated CsPbBr_3_ and CsPbI_3_ Perovskite Nanocrystals

**DOI:** 10.3390/nano16010076

**Published:** 2026-01-05

**Authors:** Ming Mei, Minju Kim, Sang Hyuk Park, Ga Eul Choi, Songyi Lee, Robert A. Taylor, Wei Chen, Suck Won Hong, Kwangseuk Kyhm

**Affiliations:** 1 Department of Cogno-Mechatronics Engineering, College of Nanoscience and Nanotechnology, Pusan National University, Busan 46241, Republic of Korea; mingmei@pusan.ac.kr (M.M.); minjukim108@pusan.ac.kr (M.K.); gecautumn@gmail.com (G.E.C.); 2 Department of Life Sciences, Faculty of Natural Sciences, Imperial College London, London SW7 2AZ, UK; sanghyuk.park@imperial.ac.uk; 3 Department of Chemistry, Pukyong National University, Busan 48513, Republic of Korea; slee@pknu.ac.kr; 4 Department of Physics, University of Oxford, Oxford OX1 3PU, UK; robert.taylor@physics.ox.ac.uk; 5 Center for Intense Laser Application Technology, College of Engineering Physics, Shenzhen Technology University, Shenzhen 518118, China; chenwei@sztu.edu.cn

**Keywords:** perovskite nanocrystal, stability, temperature dependence, band gap shift, thermal lattice expansion, electron–phonon interaction, non-radiative process

## Abstract

The temperature-dependent photoluminescence of CsPbBr_3_/SiO_2_ and CsPbI_3_/SiO_2_ nanocrystals was investigated to understand the thermal stability of SiO_2_ encapsulation. At increased temperature, intensity quenching, linewidth broadening, energy level shift, and decay dynamics were evaluated as quantified parameters. Comprehensive analysis of these parameters supports that CsPbI_3_/SiO_2_ nanocrystals show a stronger interaction with phonons compared with CsPbBr_3_/SiO_2_ nanocrystals. Despite SiO_2_ encapsulation, we conclude that trapping states are still present and the degree of localization can be characterized in terms of short-lived decay time and thermal activation energy.

## 1. Introduction

Lead halide perovskites have emerged as promising, high-performance semiconductor materials for optoelectronic applications such as solar cells [1,2,3], photodetectors [3,4,5], light-emitting diodes (LEDs) [3,6,7], lasers [8,9,10], and photocatalysts [11,12]. Because of their remarkable properties, including high photoluminescence (PL) quantum yield, narrow emission linewidth, long carrier diffusion length, and tunable band gap, they have been widely investigated for next-generation device applications. In particular, all inorganic CsPbX_3_ perovskites (X = Cl^−^, Br^−^, I^−^) have attracted considerable interest in recent years due to their robust thermal stability compared to their hybrid organic–inorganic counterparts [3,5,7]. However, CsPbX_3_ nanocrystals (NCs) still show structural, interfacial, and environmental degradation due to their soft ionic lattices and strong interactions with moisture and polar solvents [3,13,14]. In order to solve these problems, considerable effort has been devoted to improving stability such as compositional or surface engineering, matrix, and device encapsulation. In particular, the encapsulation of perovskite NCs with an appropriate inorganic protective layer such as SiO_2_ [14,15,16,17], Cs_4_PbBr_6_ [18,19], Al_2_O_3_ [14,20], TiO_2_ [21,22], and ZrO_2_ [23,24] has been a highly effective and straightforward strategy for improving their stability. Among them, SiO_2_ coating is particularly advantageous due to its excellent barrier properties against moisture and oxygen, which are the main causes of perovskite degradation. Moreover, its optical transparency across the visible spectrum ensures that they are less vulnerable to photo-degradation, making it highly suitable for optoelectronic applications [14,16,17].

For light harvesting applications, one of the most crucial optoelectronic properties of lead halide perovskite is their optical band gap. It is well known that the direct band gap of perovskites exhibits an atypical temperature dependence [25,26,27]. The conduction band minimum (CBM) of perovskites lies in the hybridized anti-bonding orbitals of the Pb 6*p* orbitals and the outer p orbitals of halide (5*p* for I, 4p for Br, and 3p for Cl), and the valence band maximum (VBM) lies in the hybridized anti-bonding states of the Pb 6s orbitals and the same halide *p*-orbital as that for the CBM. Regarding these electronic structures, the temperature-dependent band gap shift of perovskites is explained in terms of electron–phonon interactions in the corner-sharing [PbX_6_]^4−^ octahedra [28] as well as thermal expansion.

Temperature dependence in single-crystal hybrid perovskites is also considered a crucial issue for inverse temperature crystallization and device applications [29,30,31]. Beyond encapsulation strategies for nanocrystals and polycrystalline films, single-crystal perovskites have emerged as a particularly promising materials for enhanced stability and reproducibility because the absence or high reduction of grain boundaries can suppress defect-assisted degradation mechanisms and ion-migration-related instabilities. Rapid growth routes, such as inverse temperature crystallization [29], have enabled high-quality bulk single crystals with markedly improved optoelectronic quality, motivating stability-oriented device concepts based on single-crystalline forms. More recently, low-dimensional single-crystal perovskites (i.e., 2D single-crystal hybrids) have also been actively investigated, leveraging their ordered quantum-well-like structures and improved environmental tolerance [30]. Thus, the stabilizat/ion of perovskite NCs via inorganic encapsulants can be considered a complementary approach that traces the dominant surface-mediated degradation channels specific to nanoscale systems [31].

Despite the great deal of work on SiO_2_-coated CsPbX_3_ perovskite NCs [32,33,34,35], their temperature dependence of the PL spectrum has rarely been addressed [36,37]. Among the CsPbX_3_ perovskite family, most of the work has focused on green CsPbBr_3_ due to the outstanding quantum yield and thermal stability, and recent work has analyzed the PL spectrum of CsPbBr_3_/SiO_2_ NCs for increasing temperature [36]. However, the quantified parameters were not compared with other CsPbX_3_ perovskites. The quenching effect of CsPbI_3_/SiO_2_ NCs was observed in a limited temperature range (25∼253 °C), and only intensity suppression was discussed [38,39]. The PL spectrum of CsPbI_3_/SiO_2_ NCs has never been analyzed at low temperatures, and the decay dynamics remain unknown.

In this work, we investigated the temperature-dependent PL spectrum of as-prepared CsPbBr_3_/SiO_2_ NCs and CsPbI_3_/SiO_2_ NCs, which both show enhanced stability compared with the uncoated bare NCs. As the temperature increased from 4 K to 300 K, the PL spectrum was interpreted comprehensively by comparing intensity quenching, linewidth broadening, energy level shift, and decay dynamics with quantified parameters. These results enabled us to evaluate the trapped states, and the atypical temperature dependence was explained via thermal exciton dissociation, lattice expansion, and exciton–phonon interaction.

## 2. Experimental Section

### 2.1. Material Preparation and Synthesis

We used the commercial products of Cesium carbonate (Cs_2_CO_3_, reagent Plus, 99%), 1-octadecene (ODE, technical grade, 90%), oleic acid (OA, technical grade, 90%), oleylamine (OAm, technical grade, 70%), toluene (anhydrous, 99.8%), (3-aminopropyl)triethoxysilane (APTES, 99%), lead iodide (PbI_2_, 99%), methyl acetate (MeAc, reagent Plus, 99%), and ethyl acetate (EtAc, ACS reagent, ≥99.5%) from SigmaAldrich (Schnelldorf, Germany), and lead bromide (PbBr_2_, 99.998% metals basis) from Alfa Aesar (Shanghai, China). All chemicals were used as purchased without further purification.

The Cs-oleate precursor was prepared by dissolving Cs_2_CO_3_ (0.18 g) and oleic acid (0.75 mL) in 1-octadesene (7.5 mL). The mixture was stirred under vacuum and degassed at 120 °C for 1 h until a clear solution was obtained. For the synthesis of CsPbBr_3_ NCs, PbBr_2_ (0.07 g), oleic acid (0.5 mL), and oleylamine (0.5 mL) were dissolved in 1-octadescene (10 mL) and degassed at 120 °C for 40 min. The reaction flask was then filled with nitrogen, and the temperature was increased to 150 °C. Subsequently, 0.5 mL of the as-prepared Cs-oleaste solution was rapidly injected. After injection, the reaction mixture was immediately quenched via cooling in an ice-water bath. CsPbBr_3_/SiO_2_ NCs were synthesized using the same procedure as that for CsPbBr_3_ NCs, except that APTES were used to substitute oleylamine. For the synthesis of CsPbI_3_ NCs, PbI_2_ (0.087 g), oleic acid (1 mL), oleylamine (1 mL), and 1-octadecene (10 mL) were added to a three-neck flask and degassed under vacuum at 100 °C for 50 min. The temperature was then raised to 140 °C under a nitrogen atmosphere, followed by the rapid injection of 0.5 mL of the Cs-oleate solution. The reaction was terminated by cooling the flask in an ice-water bath. CsPbI_3_/SiO_2_ NCs were prepared following the same procedure as that for CsPbI_3_ NCs, with APTES substituted for oleylamine.

### 2.2. Characterization

UV-Vis absorption spectra were measured using a Spectramax M5 Microplate reader (Molecular Devices, Sunnyvale, CA, USA). Transmission electron microscopy (TEM), high-resolution transmission electron microscopy (HRTEM), and energy-dispersive X-ray spectrometry (EDS) compositional mapping images were also taken using a field emission transmission electron microscope (FETEM) operated at an accelerating voltage of 200 kV. Fourier transform infrared (FTIR) spectra were obtained. The temperature-dependent PL spectrum was obtained usinga closed-cycled cryostat (CCR), and time-resolved PL (TRPL) decay profiles were obtained using a time-correlated single photon counting (TCSPC) system with an instrument response function (IRF) of pulsed excitation (30 ps).

## 3. Results and Discussion

**Figure 1:**
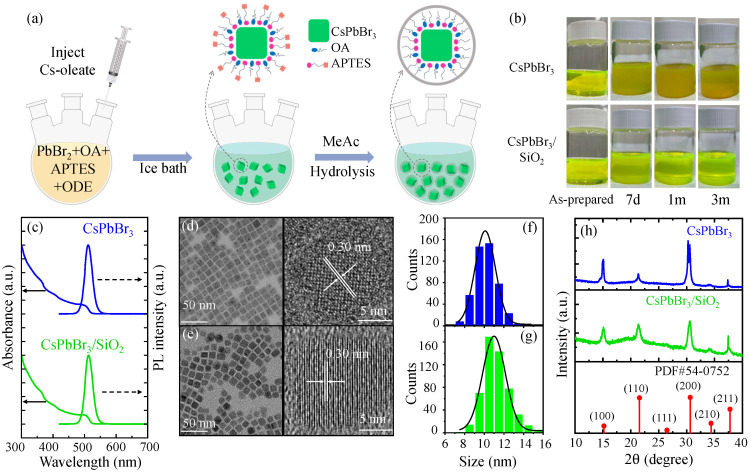
(**a**) Schematic of the synthesis process of CsPbBr_3_/SiO_2_ NCs. (**b**) As-prepared CsPbBr_3_ and CsPbBr_3_/SiO_2_ toluene solution under day light following preservation in a refrigerator at 4 °C for 0 days, 7 days, 30 days (1 month), and 3 months. (**c**) PL and absorption spectrum of CsPbBr3 and CsPbBr_3_/SiO_2_. Transmission electron microscopy (TEM) images of CsPbBr_3_ NCs (**d**) and CsPbBr_3_/SiO_2_ NCs (**e**), and the corresponding high-resolution transmission electron microscopy (HRTEM) images are also shown. (**g**) The size distribution histograms of CsPbBr_3_ NCs (**f**) and CsPbBr_3_/SiO_2_ NCs. (**h**) X-ray diffraction (XRD) patterns of CsPbBr_3_ (blue) and CsPbBr_3_/SiO_2_ NCs (green) compared with the standard pattern for cubic CsPbBr_3_ (PDF#54-0752).

Figure 1a shows the three main processes involved in synthesizing CsPbBr_3_/SiO_2_ nanocrystals (NCs). Monodisperse CsPbBr_3_/SiO_2_ NCs are prepared using a modified one-pot hot-injection approach, where (3-aminopropyl) triethoxysilane (APTES) is used as a substitution for oleylamine (OAm) and acted as the capping agent and SiO_2_ shell precursor [15,32,34]. With this method, the Cs-oleate precursor solution was quickly injected into a preheated mixture of PbBr_2_, oleic acid (OA), APTES, and 1-octadecene (ODE) at 150 °C in a three-neck flask. Upon injection, CsPbBr_3_ NCs nucleate and grow rapidly in solution. The reaction mixture is rapidly cooled to room temperature in order to stop further crystal growth and stabilize the NCs using an ice-water bath. For purification, the reaction mixture was washed with methyl acetate (MeAc), facilitating the interaction between APTES and the surface of the perovskite nanocrystals. Following this step, APTES underwent hydrolysis in the presence of moisture, where its ethoxy (-SiOC_2_H_5_) groups hydrolyzed into silanol (-SiOH) groups. Then, SiOH reacted with SiOC_2_H_5_ and/or other SiOH to form Si-O-Si cross-linked networks. This process resulted in the encapsulation of the NCs within a protective silica matrix, which enhanced their stability against environmental degradation [34]. For the synthesis of CsPbI_3_/SiO_2_ NCs, we followed a similar procedure with PbI_2_ substituting PbBr_2_. Minor variations, such as synthesis temperature and the number of APTES, were previously mentioned in the Section 2.

As shown in Figure 1b, photographs of CsPbBr_3_ and CsPbBr_3_/SiO_2_ NC solutions in toluene were taken to monitor their degradation over time. Both of the NC solutions were stored in a refrigerator at 4 °C. It is evident that both fresh perovskite solutions were transparent with a yellow-green color. However, after 7 days, the CsPbBr_3_ NC solution became opaque with significant aggregation. In contrast, the CsPbBr_3_/SiO_2_ solution remained transparent. Furthermore, after 30 days (1 month) and 3 months, the CsPbBr_3_/SiO_2_ solution was still transparent, while the CsPbBr_3_ solution continued to aggregate over time. The improved stability of CsPbBr_3_/SiO_2_ NCs can be attributed to the presence of the SiO_2_ shell, which passivates the perovskite NCs from the surface dangling bonds [16]. As a result, aggregation is suppressed, and solution stability is maintained over a long period.

Figure 1c shows the PL and absorption spectra of CsPbBr_3_ NCs and CsPbBr_3_/SiO_2_ NCs, where the central PL wavelengths and linewidth (FWHM) of CsPbBr_3_/SiO_2_ NCs and CsPbBr_3_ NCs are observed to be 513 nm/511 nm and 28.4 nm/27.8 nm, respectively. A uniform size distribution was observed in both TEM images of CsPbBr_3_ NCs (Figure 1d) and CsPbBr_3_/SiO_2_ NCs (Figure 1e), and the HRTEM images also show a clear lattice spacing of 0.30 nm, which corresponds to the (200) plane of cubic-phase CsPbBr_3_ [40]. These results indicate that the crystal integrity of CsPbBr_3_ NCs is well preserved after silica coating. Because the shapes of both CsPbBr_3_ NCs and CsPbBr_3_/SiO_2_ NCs are rectangular rather than square, the diagonal length of the rectangles was measured to determine the size distribution. In Figure 1f and Figure 1g, the diagonal size distribution histograms of CsPbBr_3_ NCs (10.2 ± 1.1 nm) and CsPbBr_3_/SiO_2_ NCs (11.3 ± 1.4 nm) are shown, respectively. Recent work observed a slight size expansion when CsPbBr_3_ NCs are coated with SiO_2_ [36,37]. Although the increased size causes a redshift as a result of the decreased confinement energy, the energy difference between CsPbBr_3_ NCs and CsPbBr_3_/SiO_2_ NCs is barely seen.

To confirm the crystal structure of CsPbBr_3_ NCs and CsPbBr_3_/SiO_2_ NCs, their X-ray diffraction (XRD) patterns were analyzed and compared with the standard PDF card, as shown in Figure 1h. The CsPbBr_3_/SiO_2_ NCs exhibit strong diffraction peaks at 2θ = 15.2°, 21.5°, 30.8°, 34.5°, and 37.8°, corresponding to the planes of (100), (110), (200), (210), and (211) in cubic CsPbBr_3_ (PDF#54-0752) [35], respectively. It is also noticeable that no structural phase transition was observed after silica shell coating, and this result indicates that the crystal integrity of CsPbBr_3_ NCs was well preserved. These XRD results are consistent with the high-resolution TEM images shown in the insets of Figure 1d,e, which confirm the structural stability of the CsPbBr_3_/SiO_2_ NCs. Although the diffracted peak intensity of CsPbBr_3_/SiO_2_ NCs appears decreased compared with that of bare CsPbBr_3_ NCs, this is mainly attributed to the incorporation of an amorphous SiO_2_ fraction, which dilutes the crystalline perovskite content and increases diffuse background scattering. It is important that the unchanged peak positions and the absence of additional reflections confirm that the cubic CsPbBr_3_ crystal phase was preserved after encapsulation.

The atomic-resolution scanning transmission electron microscopy (STEM) images, which were acquired in a high-angle annular dark field (HAADF) mode (Figure S1 in the Supplementary Materials), revealed that CsPbBr_3_ NCs were embedded within the silica shell. Additionally, the soft edges observed in the TEM images of CsPbBr_3_/SiO_2_ NCs further indicated the presence of an ultrathin SiO_2_ shell [26]. The Fourier transform infrared (FTIR) spectrum (Figure S2 in the Supplementary Materials) provided additional evidence for the core–shell structure of CsPbBr_3_/SiO_2_ NCs. The sharp peak at approximately 1128 cm^−1^ was attributed to the stretching vibration of the Si–O–Si bond [26]. Moreover, the peak at around 1033 cm^−1^ corresponded to the vibration of the Si–O–C bond. The detected peaks at approximately 2854 cm^−1^ and 2923 cm^−1^ were assigned to the asymmetric and symmetric stretching vibrations of C–H, respectively.

As demonstrated in the case of CsPbBr_3_, SiO_2_ encapsulation contributed to improved stability. Given these promising results, CsPbI_3_ NCs were also encapsulated with SiO_2_ to examine whether a similar stabilization effect could be achieved. It is known that the ammonium ligands of CsPbI_3_ NCs are easily lost due to the weak acid–base interactions between I and oleylammonium, resulting in fast agglomeration and an undesired phase transformation from cubic to orthorhombic. Their thermodynamically unstable α-phase also causes rapid degradation. To revolve these unstable issues in CsPbI_3_ NCs, many strategies were known to improve its stability such as introducing organolead compound trioctylpho sphine-PbI_2_ (TOP-PbI_2_) as the reactive precursor [41], utilizing ZnI_2_ as a co-precursor and passivating agent in the synthesis [42].

**Figure 2:**
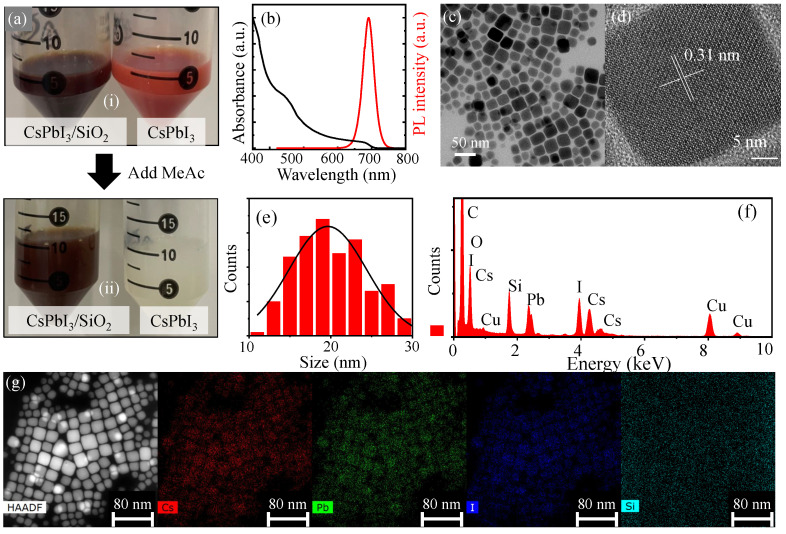
(**a**) A total of 5 mL of as-prepared CsPbI_3_ and CsPbI_3_/SiO_2_ pristine solution (**i**), and the two pristine perovskite solution after adding 5 mL methyl acetate into 5 mL (**ii**), where the insets are photographs of CsPbI_3_/SiO_2_ toluene solution under day light, respectively. (**b**) PL and absorption spectra of CsPbI_3_/SiO_2_ NCs. Given TEM (**c**) and HRTEM (**d**) images of CsPbI_3_/SiO_2_ NCs, the size distribution histogram was obtained (**e**) as well as the lattice spacing. (**f**) The energy-dispersive X-ray (EDX) spectrum of CsPbI_3_/SiO_2_ NCs. (**g**) For an STEM-HAADF image of CsPbI_3_/SiO_2_ NCs, the elemental mapping images of Cs, Pb, I, and Si were compared.

As shown in Figure 2a, two solutions were prepared: a brown-colored CsPbI_3_/SiO_2_ NC solution and a burgundy-colored pristine CsPbI_3_ NC solution. To remove excess ligands and byproducts from the reaction, both solutions underwent a purification step using methyl acetate. During this process, the pristine CsPbI_3_ NC solution was found to show rapid degradation and changed color from burgundy to white, indicating structural instability. In contrast, the CsPbI_3_/SiO_2_ NC solution retained its original brown color, demonstrating that the SiO_2_ shell effectively prevented degradation (Figure S6 in the Supplementary Materials). However, CsPbI_3_ NCs exhibited a distinct behavior compared to CsPbBr_3_ NCs, undergoing rapid PL quenching upon purification. This inhibits comparative studies on their optical properties.

The stability of CsPbI_3_/SiO_2_ NCs was further confirmed, as no noticeable quenching was observed over time. To investigate their optical properties, the absorption and PL spectra of CsPbI_3_/SiO_2_ NCs were measured as shown in Figure 2b. The absorption spectrum exhibited two prominent peaks near 680 nm and 450 nm. The PL spectrum showed a peak centered around 690 nm with a full width at half maximum (FWHM) of 39 nm, which is broader than that of CsPbBr_3_/SiO_2_ NCs (28.4 nm). The relatively narrow FWHM value also indicates a uniform size distribution of CsPbI_3_/SiO_2_ NCs. This observation was further supported by the TEM image in Figure 2c, which visually confirms the uniform morphology of the nanocrystals. The HRTEM image in Figure 2d also shows clear lattice fringes of CsPbI_3_/SiO_2_ NCs, indicating their high crystalline quality. The measured lattice spacing distance of 0.31 nm corresponds to the (200) planes of cubic-phase CsPbI_3_, further confirming the structural integrity of the encapsulated NCs. The corresponding size distribution analysis reveals that the CsPbI_3_/SiO_2_ NCs have an average size of approximately 20.3 nm with a standard deviation of ±4.4 nm, as shown in Figure 2e.

Figure 2f shows the energy-dispersive X-ray (EDX) spectrum of CsPbI_3_/SiO_2_ NCs, while Figure 2g displays the STEM-HAADF image and elemental mapping images, confirming the successful formation of the silica shell around CsPbI_3_/SiO_2_ NCs. For further verification of a core–shell structure, Fourier transform infrared (FTIR) spectroscopy was performed (Figure S2 in Supplementary Materials), revealing strong peaks at 1037 cm^−1^ and 1070 cm^−1^, which correspond to the stretching vibrations of Si-O-Si [39] and Si-O-C [43] bonds, respectively. To assess the effect of the silica shell on the crystal structure of CsPbI_3_/SiO_2_ NCs, X-ray diffraction (XRD) measurements were conducted and compared with the reference pattern from ICSD no. 181288 (Figure S4 in the Supplementary Materials). The XRD peaks of CsPbI_3_/SiO_2_ NCs closely match those of ICSD no. 181288 within the range of 2θ = 10 °–40°, confirming that CsPbI_3_/SiO_2_ NCs retain a cubic phase.

**Figure 3:**
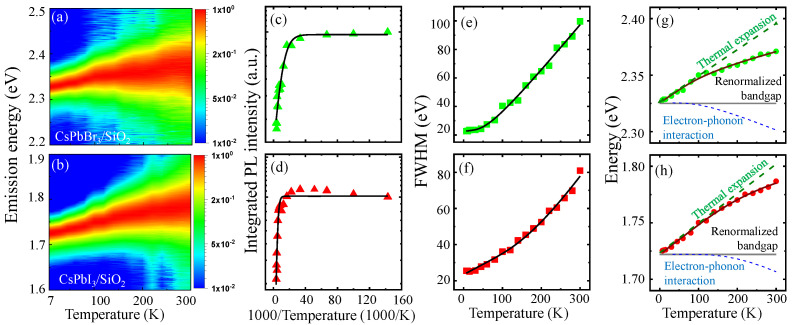
(**a**,**b**) Given the temperature-dependent PL spectrum of CsPbBr_3_/SiO_2_ and CsPbI_3_/SiO_2_ NCs, which are normalized to the peak intensity for clarity, spectrally integrated PL intensity (**c**,**d**), linewidth (**e**,**f**), and peak energy (**g**,**h**) are plotted with regard to temperature, respectively.

Although the temperature-dependent PL spectrum in perovskites NCs was extensively investigated [25,26,27,28,36,37,38], a systematic study of the core–shell perovskite NCs is still necessary. In Figure 3a,b, the PL intensity of CsPbBr_3_/SiO_2_ NCs and CsPbI_3_/SiO_2_ NCs was obtained for spectrum and temperature, respectively. The integrated PL intensity of CsPbBr_3_/SiO_2_ NCs (Figure 3c) and CsPbI_3_/SiO_2_ NCs (Figure 3d) is also shown for reciprocal temperature (1000/T) to evaluate thermal activation. With increasing temperature, thermally activated non-radiative recombination processes, leading to a gradual decrease in PL intensity. For quantitative evaluation, a model with the Arrhenius equation was utilized [26]:

(1) $$I\left(T\right)=\frac{I_{0}}{1+A\exp(-\frac{E_{A}}{k_{B}T})}\;$$

where I(T) and $I_{0}$ are the integrated PL intensity at temperature *T* and 0 K, respectively. Regarding the relative intensity quenching with $I_{0}/I\left(T\right)=1+A\exp\left(-E_{A}/k_{B}T\right)$, it is plausible that an activation energy $E_{A}$ in the Boltzmann factor can be obtained with a fitting constant *A*, which is associated with thermal dissociation. In the case of CsPbI_3_/SiO_2_, a high $E_{A}=63.8\pm 11.9$ meV was obtained, which is comparable to its exciton binding energy. This result suggests that thermal exciton dissociation is a dominant mechanism of PL quenching. Moreover, a low $E_{A}=14.9\pm 1.5$ meV was obtained in CsPbBr_3_/SiO_2_ NCs. Therefore, the presence of additional non-radiative processes is expected, such as trap-assisted carrier recombination, exciton delocalization, and phonon-mediated scattering. Hence, CsPbBr_3_/SiO_2_ NCs are vulnerable to non-radiative losses, and this may explain the significant difference between Figure 3a,b.

Enhanced phonon scattering for temperature leads to a linewidth broadening of the PL spectrum, and this can be described by the following equation [25,26]:

(2) $$\Gamma \left(T\right)=\Gamma_{0}+\sigma T+\frac{\Gamma_{\mathrm{op}}}{\exp(E_{\mathrm{op}}/k_{B}T)-1}\;$$

where $\Gamma_{0}$ is the inhomogeneous linewidth independent of temperature, caused by variations in the size, shape, and composition of NCs. The linear term (σT) is attributed to the acoustic–phonon interaction with the acoustic–phonon coupling coefficients (σ). The third nonlinear term is described in terms of the coupling coefficient ($\Gamma_{\mathrm{op}}$) and the energy ($E_{\mathrm{op}}$) of optical phonons, which are associated with the Bose–Einstein distribution.

In Figure 3e,f, the temperature-dependent linewidth of CsPbBr_3_/SiO_2_ NCs and CsPbI_3_/SiO_2_ NCs is fitted with Equation (2), where the fitting parameters are summarized in Table S1 of the Supplementary Materials. With increasing temperature from 7 K to 300 K, the linewidth becomes broadened by 76.96 meV and 55.43 meV in CsPbBr_3_/SiO_2_ NCs and CsPbI_3_/SiO_2_ NCs, respectively. The inhomogeneous linewidths of CsPbBr_3_/SiO_2_ ($\Gamma_{0}=22.48\pm 1.91$ meV) and CsPbI_3_/SiO_2_ ($\Gamma_{0}=23.41\pm 0.927$ meV) are significantly narrow compared to the recent results of uncoated CsPbBr_3_ NCs (40.04 ± 0.45 meV) and CsPbI_3_ NCs (52.12 ± 0.71 meV). This result indicates that the size homogeneity and surface passivation are improved in CsPbBr_3_/SiO_2_ NCs and CsPbI_3_/SiO_2_ NCs. We found that the optical phonon interaction of the third term is dominant compared with the acoustic interaction of the second term (∼σT), and the $E_{\mathrm{LO}}$ of CsPbBr_3_/SiO_2_ (15.516 ± 6.102 meV) and CsPbI_3_/SiO_2_ (55.106 ± 17.151 meV) were comparable to the LO phonon energy [36].

As temperature increases, the band gap shift can be observed. The renormalized band gap E_g_(T) can be described by the following equation [25,27]:

(3) $$E_{g}\left(T\right)=E_{0}+A_{T}T+A_{P}\left[\frac{2}{\exp(E_{P}/k_{B}T)-1}+1\right]$$

where the thermal lattice expansion effect results in a linear dependence for temperature (∼$A_{T}T$) with the thermal expansion coefficient $A_{T}$, and the electron–phonon interaction of the third term leads to a nonlinear temperature dependence with the phonon coupling strength $A_{P}<0$ and the average optical phonon energy $E_{P}$. Given the band gap $E_{0}$ at T=0K, the thermal lattice expansion effect causes a blueshift of the band gap, but the electron–phonon interaction gives rise to an opposite redshift. Given the PL peak energy of CsPbBr_3_/SiO_2_ NCs (Figure 3g) and CsPbI_3_/SiO_2_ NCs (Figure 3h), Equation (3) was fitted (solid line), and the parameters are shown in Table S2 of the Supplementary Materials. This model enables us to separate the two effects of thermal lattice expansion and electron–phonon interaction (dotted lines). At low temperatures, the band gap shift is dominated by the thermal lattice expansion. In the case of typical semiconductors, the thermal lattice expansion results in a band gap decrease ($A_{T}<0)$. In contrast, we obtained positive $A_{T}$ for CsPbI_3_/SiO_2_ (0.23 ± 0.03 meV/K) and CsPbBr_3_/SiO_2_ (0.26 ± 0.02 meV/K). In the case of perovskite (CsPbX_3_ NCs, the anti-bonding nature of the interaction between Pb 6*s* and halide *p* orbitals becomes significant, and this leads to a distinctive thermal expansion effect of the [PbX_6_]^4−^ octahedral framework.

At high temperatures, sufficient thermal energy excites large energy phonon states, and the Bose–Einstein distribution in the third term of Equation (2) becomes significant due to the activated optical phonon modes. This effect was quantified in terms of the phonon coupling strength ($A_{P}$) and the average optical phonon energy ($E_{P}$). A large $A_{P}=93.3\;$meV was obtained in CsPbI_3_/SiO_2_ compared with the small $A_{P}=25.3\;$meV in CsPbBr_3_/SiO_2_. The large $E_{P}$ in CsPbI_3_/SiO_2_ (66.6 meV) can also explain why the electron–phonon interaction effect remains suppressed at low temperatures. However, the small $E_{P}$ in CsPbBr_3_/SiO_2_ (29.3 meV) provides a relatively lower temperature threshold to activate optical phonons.

**Figure 4:**
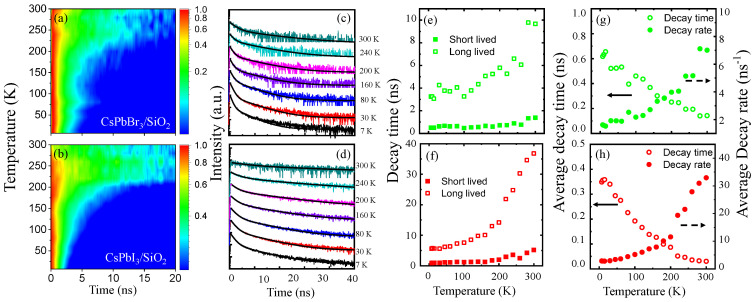
(**a**,**b**) Given time-resolved PL intensity of CsPbBr_3_/SiO_2_ and CsPbI_3_/SiO_2_ NCs for increasing temperature, the PL intensity decay were fitted by the bi-exponential model at various temperatures (**c**,**d**), respectively. This enables us to obtain short- and long-lived decay time for temperature (**e**,**f**), whereby the corresponding average decay time and average decay rate were also obtained for temperature (**g**,**h**).

In Figure 4, the time-resolved PL intensity of CsPbBr_3_/SiO_2_ NCs (a,c) and CsPbI_3_/SiO_2_ NCs (b,d) is plotted for temperature, which were selected at the dominant PL energy, respectively. For quantitative analysis, two kinds of decay times in CsPbBr_3_/SiO_2_ NCs (Figure 4e,g) and CsPbI_3_/SiO_2_ NCs (Figure 4f,h) were obtained at various temperatures using a bi-exponential model,

(4) $$E_{g}\left(T\right)=A_{1}\exp\left(-t/\tau_{1}\right)+A_{2}\exp\left(-t/\tau_{2}\right)$$

where $\tau_{1}$ and $\tau_{2}$ are the short- and long-lived decay time, and $A_{1}$ and $A_{2}$ are the corresponding amplitudes, respectively. For increasing temperature, we found that $\tau_{1}$ barely changes with decreased $A_{1}$. However, both $\tau_{2}$ and $A_{2}$ show a significant increase for temperature. These results are associated with thermally activated carriers and phonons. At low temperatures, excited carriers are initially trapped in localized states. However, thermal activation suppresses the non-radiative trapping. Exciton recombination time can also be elongated as the interaction with phonons becomes pronounced. While the former mechanism alters the relative weight of $\tau_{1}$ and $\tau_{2}$, the latter enhances the dark exciton states and the exciton dissociation. Recent work has reported a similar evidence in the strong exciton–phonon coupling in CsPbX_3_ perovskite structures [44]. Interestingly, for increasing temperature from 7 K to 300 K, $\tau_{2}$ of CsPbBr_3_/SiO_2_ NCs increases from 4ns to 10ns, but the change in $\tau_{2}$ in CsPbI_3_/SiO_2_ NCs is more significant: 40 ns at 300 K. This result is also consistent with the high $A_{P}=93.3\;$meV of CsPbI_3_/SiO_2_ NCs compared with that of CsPbBr_3_/SiO_2_ NCs ($A_{P}=25.3\;$ meV).

To obtain a representative quantity of the decay feature, the average decay time ($\tau_{\mathrm{avg}}$) was also obtained using the four parameters in the bi-exponential model as

(5) $$\tau_{\mathrm{avg}}=\frac{A_{1}\tau_{1}^{2}+A_{2}\tau_{2}^{2}}{A_{1}\tau_{1}+A_{2}\tau_{2}}.$$

In Figure 4g,h, the $\tau_{\mathrm{avg}}$ and 1/$\tau_{\mathrm{avg}}$ of CsPbBr_3_/SiO_2_ NCs and CsPbI_3_/SiO_2_ NCs are shown for increasing temperature, respectively. Compared with bare CsPbBr_3_ NCs, CsPbBr_3_/SiO_2_ NCs are known to show a prolonged PL decay time [36]. The overall feature was correct, but the monotonic model was not enough to define a representative decay time. It is noticeable that the amplitudes ($A_{1}$ and $A_{2}$) were also considered to obtain the average decay time. At low temperatures, $1/\tau_{\mathrm{avg}}$ shows a gradual increase, but a dramatic increase occurs at a critical temperature ($\tau_{c}∼200\;$K). This critical phenomenon is significant in CsPbI_3_/SiO_2_ NCs and is consistent with the strong phonon interactions with the high $A_{P}$ and $E_{P}$. Therefore, $\tau_{c}$ is useful to evaluate the degree of localization and thermal activation.

As an additional remark, CsPbI_3_/SiO_2_ NCs can be an attractive material system for optical devices. In particular, they are suitable for LED and phosphor-converted color conversion, where their band gap tunability and narrow emission linewidths enable spectrally pure red emission for wide-color-gamut displays and high-brightness solid-state lighting. At the same time, these favorable optical characteristics are also useful in photodetector and photo-conductive architectures, in which strong absorption coefficients and well-defined band edges facilitate efficient photo-carrier generation. Nevertheless, these applications are constrained by temperature-activated non-radiative recombination pathways and phase instability, which are often exacerbated under operational conditions. In this context, it is important to understand how encapsulation-induced stabilization translates into temperature-resilient optical behavior.

## 4. Conclusions

Compared with the bare NCs of CsPbBr_3_ and CsPbI_3_, we confirmed that SiO_2_ encapsulation improves the stability of the core–shell structures. Although the core crystal structures remain unaffected in the presence of a SiO_2_ shell, we found that trapping states are still present. Their degree of localization can be quantified in terms of a short-lived decay time and thermal activation energy, as well as the vibration modes of the Si–O–Si and Si–O–C bonds. From our comprehensive analysis of the intensity, linewidth, energy level shift, and decay time of PL spectrum at increased temperature, we concluded that CsPbI_3_/SiO_2_ NCs show a stronger interaction with phonons compared with CsPbBr_3_/SiO_2_ NCs.

## Data Availability

The data presented in this study are available on request from the corresponding author.

## Supplementary Materials

The following supporting information can be downloaded at: https://www.mdpi.com/article/10.3390/nano1010000/s1, Figure S1: STEM-HAADF image for CsPbBr_3_/SiO_2_; Figure S2: FTIR spectrum; Figure S3: The as-prepared CsPbBr_3_ and CsPbBr_3_/SiO_2_ toluene solution under day light; Figure S4: XRD pattern; Figure S5: The as-prepared CsPbI_3_ and CsPbI_3_/SiO_2_ toluene solution; Figure S6: Visualization of RGB color evolution of CsPbI_3_ and CsPbI_3_/SiO_2_ toluene solution; Table S1: Fitting parameters for PL FWHM; Table S2: Fitting parameters for PL peak energy.
