# Supplementary material for: Comprehensive Analysis of Temperature-Dependent Photoluminescence in Silica-Encapsulated CsPbBr3 and CsPbI3 Perovskite Nanocrystals
Source: Nanomaterials (Basel). 2026 Jan 5;16(1):76. doi: 10.3390/nano16010076 (PMC12787875; doi:10.3390/nano16010076)
Supplement: Supplementary file 1 [file nanomaterials-16-00076-s001.zip › nanomaterials-4057610-supplementary.pdf]

Supplementary Materials

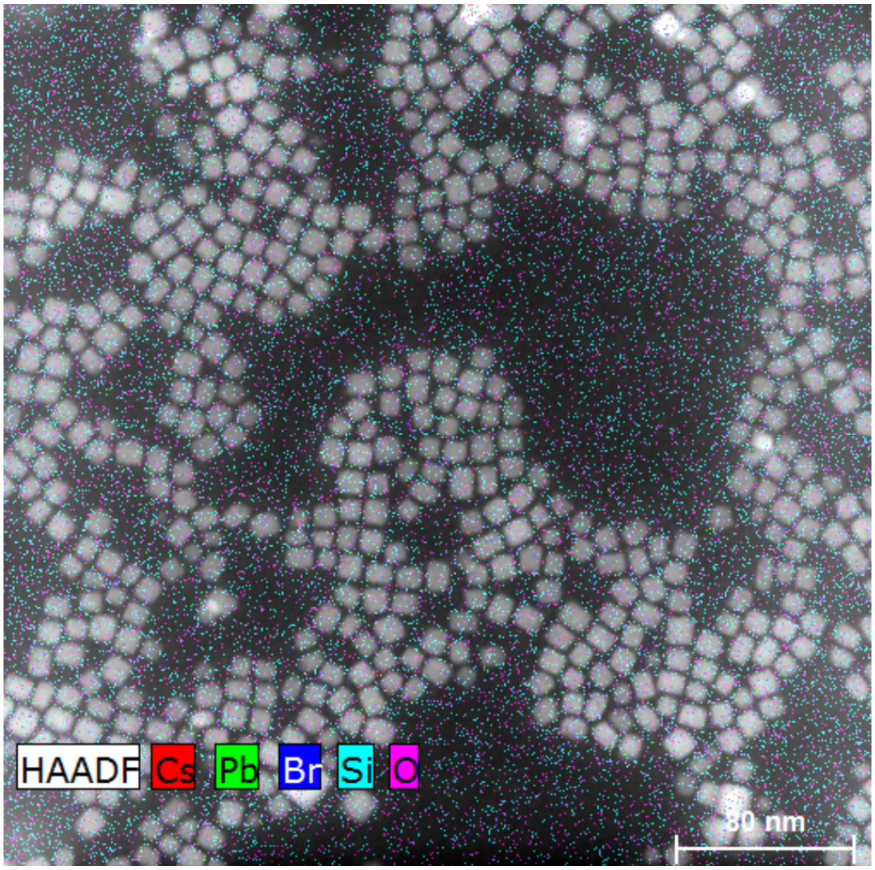

Figure S1. STEM-HAADF image and elemental mapping image showing the elemental distribution of Cs, Pb, Br, Si and O of the $CsPbBr_3@SiO_2$ NCs

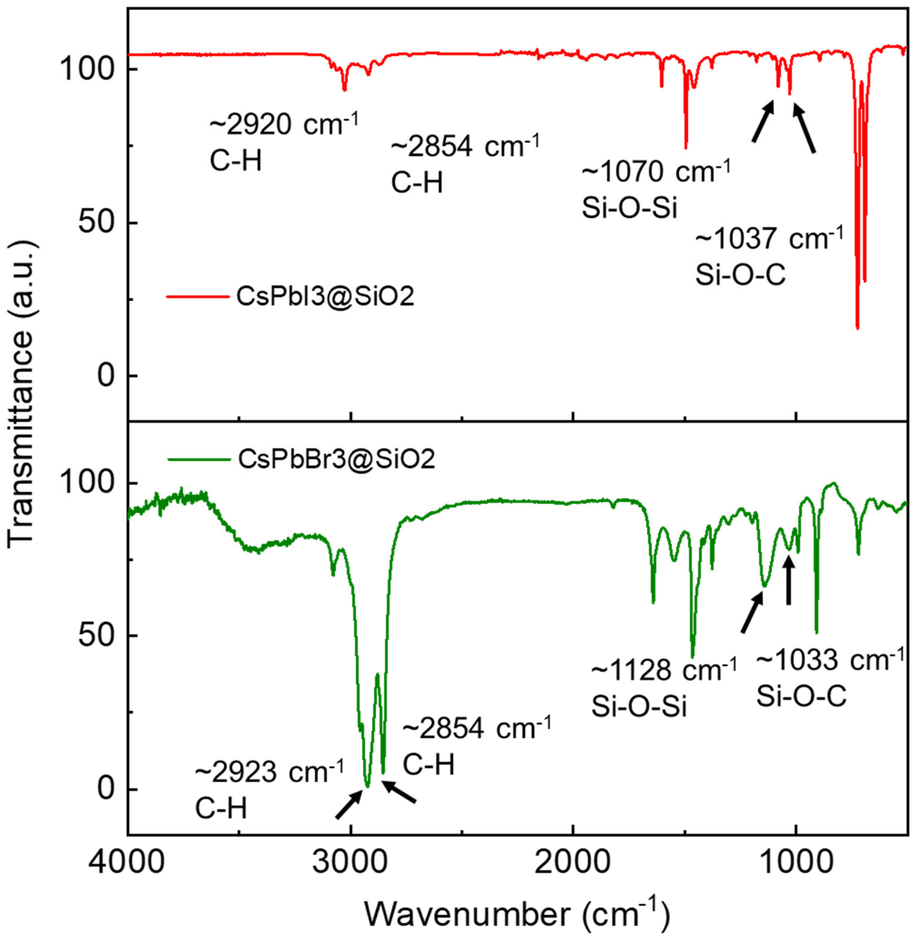

Figure S2. FTIR spectrum of as-prepared $CsPbBr_3/SiO_2$ and $CsPbI_3/SiO_2$ NCs.

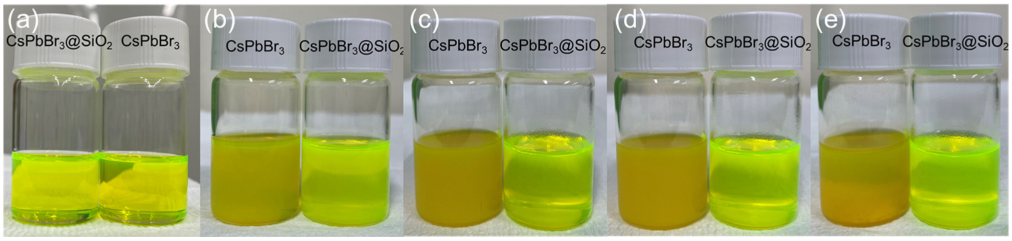

Figure S3. The as-prepared $CsPbBr_3$ and $CsPbBr_3/SiO_2$ toluene solution under day light after preserved in the refrigerator at 4 °C for 0 day (a), 7 days (b), 19 days (c), 30 days (1 month) (d) and 3 months (e).

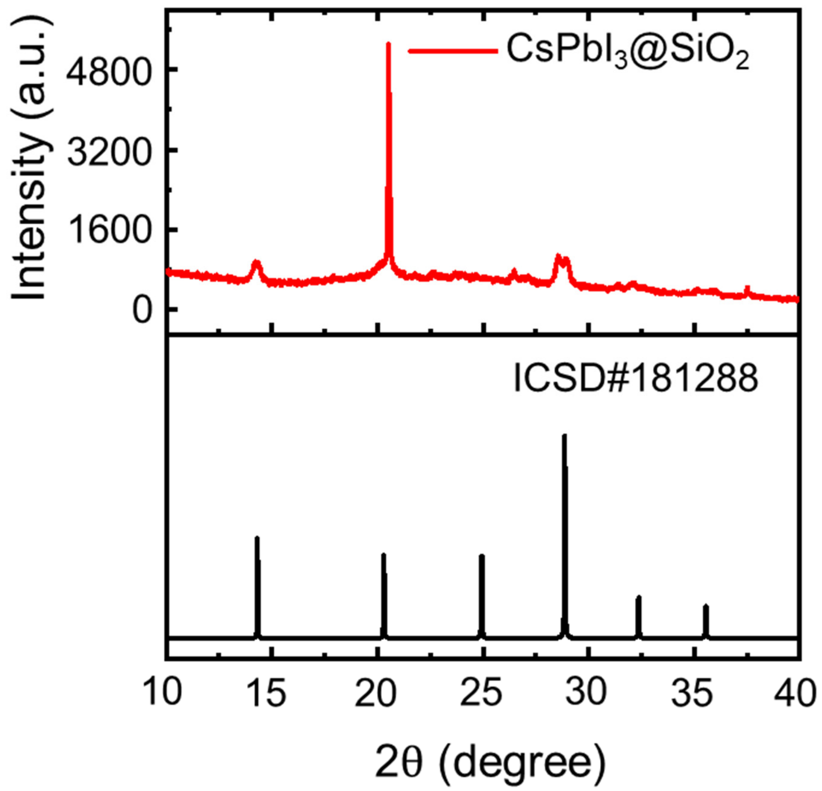

Figure S4. XRD pattern of $CsPbI_3/SiO_2$ NCs (red) and the standard pattern for cubic $CsPbI_3$ (ICSD#181288).

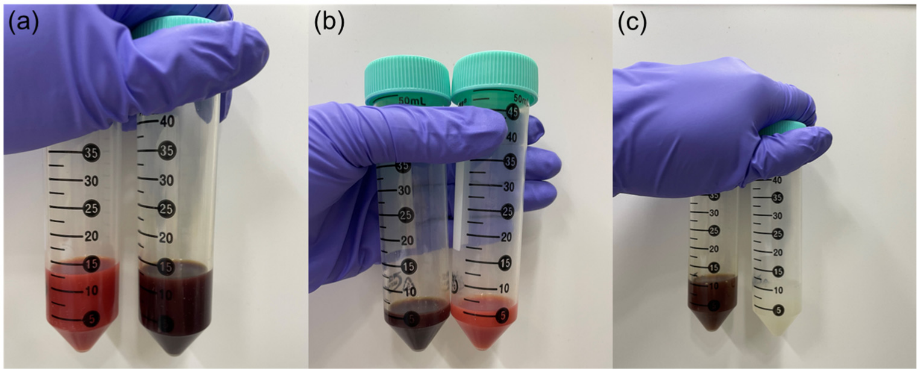

Figure S5. The as-prepared $CsPbI_3$ and $CsPbI_3/SiO_2$ pristine solution (a), 5 ml of the two pris tine perovskite solution, and after adding 5 ml methyl acetate into 5 ml of the two pristine per ovskite solution (c).

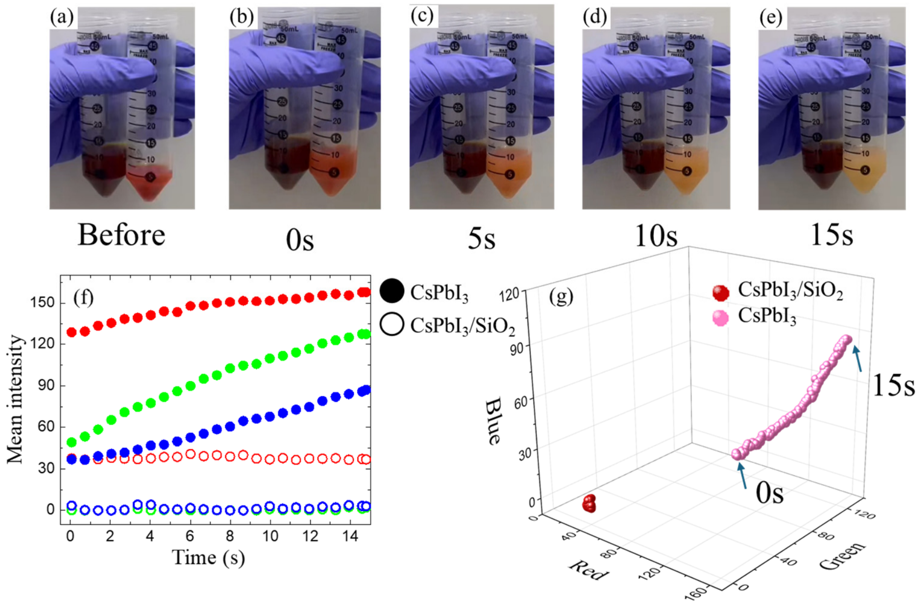

Figure S6. Photographic images showing the RGB color evolution of $CsPbI_3$ and $CsPbI_3/SiO_2$ pristine solutions upon the addition of 5 mL of methyl acetate. (a) Before methyl acetate addition, and after addition at (b) 0 s, (c) 5 s, (d) 10 s, and (e) 15 s, respectively. (f) Quantitative analysis of the color change represented by the extracted RGB values as a function of time. (g) Three-dimensional visualization of the RGB coordinates illustrating the trajectory of color evolution in RGB color space.

Table S1. Parameters derived via fitting the PL FWHM temperature dependence with equatio n (2)

Perovskite NCs	Γ_0 (meV)	σ (eV/K)	Γ_{op} (meV)	E_{op} (meV)
$CsPbBr_3/SiO_2$	22.48±1.91	$3.5586 * 10^{-5} \pm 8.832 * 10^{-5}$	52.76±11.62	15.516±6.102
$CsPbI_3/SiO_2$	23.41±0.927	$1.1406 * 10^{-4} \pm 1.66158 * 10^{-5}$	150.14±91.99	55.106±17.151

Table S2. Parameters derived via fitting the PL peak energy temperature dependence using equat ion (5).

Perovskite NCs	E_0 (eV)	Eg(T=0) (eV)	A_T (meV/K)	A_P (meV)	E_P (meV)
$CsPbBr_3/SiO_2$	2.351± 0.011	~2.325	0.231±0.030	-25.27±10.64	29.247±11.897
$CsPbI_3/SiO_2$	1.815±0.075	~1.722	0.261±0.013	-93.29±75.07	66.584±22.340
